# Remote Training for Medical Staff in Low-Resource Environments Using Augmented Reality

**DOI:** 10.3390/jimaging8120319

**Published:** 2022-11-29

**Authors:** Austin Hale, Marc Fischer, Laura Schütz, Henry Fuchs, Christoph Leuze

**Affiliations:** 1 UNC Graphics and Virtual Reality Group, Department of Computer Science, University of North Carolina at Chapel Hill, Chapel Hill, NC 27599, USA; 2 Nakamir Inc., Menlo Park, CA 94025, USA; 3 School of Engineering, Stanford University, Stanford, CA 94305, USA; 4 Chair for Computer Aided Medical Procedures and Augmented Reality, Department of Informatics, Technical University of Munich, 80333 Munich, Germany

**Keywords:** augmented reality, virtual reality, remote collaboration, computer-aided interventions, telemedical education, teleconsultation, point cloud streaming, low-resource environments

## Abstract

This work aims to leverage medical augmented reality (AR) technology to counter the shortage of medical experts in low-resource environments. We present a complete and cross-platform proof-of-concept AR system that enables remote users to teach and train medical procedures without expensive medical equipment or external sensors. By seeing the 3D viewpoint and head movements of the teacher, the student can follow the teacher’s actions on the real patient. Alternatively, it is possible to stream the 3D view of the patient from the student to the teacher, allowing the teacher to guide the student during the remote session. A pilot study of our system shows that it is easy to transfer detailed instructions through this remote teaching system and that the interface is easily accessible and intuitive for users. We provide a performant pipeline that synchronizes, compresses, and streams sensor data through parallel efficiency.

## 1. Introduction

### 1.1. Clinical Background

Medical augmented reality (AR) is a highly scalable technology that improves the capabilities of medical caregivers by guiding medical caregivers through diagnostic and treatment protocols and projecting relevant information directly into the medical caregiver’s field of view [1,2]. Due to the rapid developments and falling costs of AR hardware, medical AR can also play an important role in helping with training and extending medical capabilities in resource-limited settings. Limited access to essential health services is a major problem in the developing world. According to World Health Organization (WHO) estimates in 2013, there was a global shortage of 7.2 million qualified healthcare workers, a number that will increase to 12.9 million by 2035 [3]. There is also a wide gap between high- and low-income countries: countries such as the USA have more than 2.5 physicians per 1000 people, while countries such as Kenya have only 0.15 physicians per 1000 people, a number that is even lower for rural areas [3]. Although medical training and medical schools require expensive infrastructure that cannot be easily deployed in these areas, most healthcare workers have access to cell phone service [4]. In Sub-Saharan Africa, cell phone ownership has increased from around 10% to 80% between 2002 and 2014 [5].

AR headsets are similar to cell phones in many respects. Like cell phones, they are essentially mobile computers with a display and do not require expensive infrastructure, which means that they can also be deployed in remote locations and small hospitals without modern medical equipment. In cases like Microsoft’s HoloLens 2, see-through AR head-mounted displays (HMDs) provide sensors and dedicated processors for simultaneous localization and mapping (SLAM) or hand tracking, which make them still considerably more expensive than cell phones. However, AR headset technology is constantly improving and prices are constantly falling, making this technology more and more accessible. Provided there are sufficiently useful applications, AR HMDs may experience similar growth in low-resource areas as cell phones in the future. For this reason, our aim is to leverage medical AR technology to counter the shortage of medical experts in low-income countries by providing a remote training process for medical procedures.

### 1.2. Related Works

Remote collaboration and guidance is an important application for AR [6]. The Microsoft HoloLens has been tested as a telemedicine platform for remote procedural training [7]. Due to the small form factor and the ability to be deployed in far-forward environments, AR has also been tested as a remote guidance tool for battlefield care [8]. Researchers at Purdue University have developed the System for Telementoring with Augmented Reality (STAR) to create a portable and self-contained telementoring platform for critical surgeries such as cricothyrotomies [9]. The ability of medical experts to remotely support clinical point-of-care providers through AR has been tested as a tool for damage control surgery even in remote settings and on the battlefield [10,11].

Previous work on remote medical training in augmented reality has explored a training setup in which the first person, the streamer, wears an HMD and the second person, the receiver, sees a rendering of the first person’s view on a screen or an HMD [12]. Researchers at UC San Diego developed a system called ARTEMIS, which enables experienced surgeons and novices to share the same virtual space: Expert surgeons at remote sites use virtual reality (VR) to access a 3D reconstruction of the patient’s body and instruct novice surgeons on complex procedures as if they were together in the operating room [13].

Similarly, the ARTEKMED research project from the Technical University of Munich is a remote surgical application that scans the entire operating room, allowing a remote physician to visualize the scanned scene through VR and advise attending physicians with AR HMDs [14]. Another study has compared representing either an avatar or a point cloud reconstruction as the remote user in AR. Their results showed that the point cloud reconstruction-based representation of the remote medical expert was rated higher in social interaction and copresence than the virtual avatar [15]. Hence, our AR remote training system uses point cloud reconstruction to visualize the remote patient. The ARTEKMED research group has also evaluated teleconsultation for the use case of a preclinical emergency in which a remote expert instructs a paramedic on emergency procedures [16].

Like the work of Strak et al., our objective is to develop an application that allows a medical expert to remotely instruct a student, specifically for medical training in low-resource environments [16]. To provide a solution that can be used with minimal infrastructure, we eliminate the need for extensive camera equipment used in other studies to scan the room of the person seeking training or consultation [14,16]. Instead, we present a standalone system that uses only one HMD in both locations to realize a remote training application for low-resource environments.

## 2. Materials & Methods

**Figure 1:**
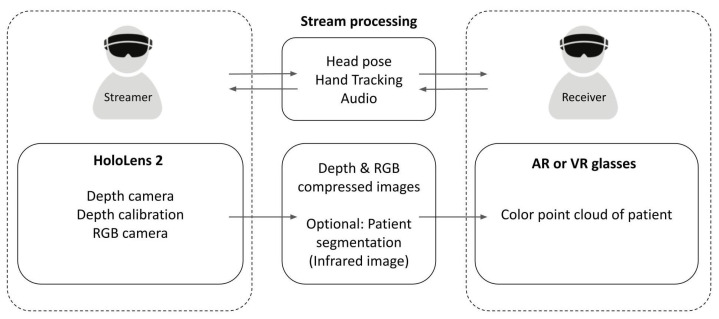
Project architecture and breakdown of the streamer and receiver into their components. By seeing the 3D patient with which the streamer is interacting, the receiver can shadow the interaction or provide feedback.

We used Microsoft’s HoloLens 2 (v22H1) and Epic Games’ Unreal Engine (v4.27) to develop a remote teaching solution for medical applications consisting of two components, a teacher application and a student application (Figure 1). Our AR application includes two modes: a streamer mode that streams the Microsoft HoloLens 2 depth and color data, and a receiver mode that receives the data from the streamer and combines color and depth to visualize a color point cloud of the streamer’s field of view on any OpenXR-compatible device.

### 2.1. Streaming Application

In our application, a HoloLens 2 user streams the color and depth camera data, as well as the HoloLens 2 pose (position and orientation) and audio to a remote AR device. The HoloLens 2 provides information about the real-world pose of the user, while the depth camera provides information about the real-world environment in front of the user, such as the patient during a remote medical procedure.

We created an application based on the HoloLens 2 Research Mode API for easy access to the depth camera sensor [17,18]. First, we calibrate the HoloLens 2 cameras to undistort the camera images. Since the Research Mode API only exposes the camera pixel data mapped to the unit plane instead of each camera sensor’s intrinsics and distortion coefficients, we iterate through each pixel and pre-compute the depth mappings for the streaming device. We then serialize and save the camera mappings to the device’s local documents folder for future retrieval. This calibration method saves time for users who start the app, since it quickly deserializes the saved mappings instead of computing them for every launch on the same device. The stream of depth images on one device must attach this metadata to accurately depict the 3D point cloud visualization on another device. Additionally, by storing these mappings beforehand, we save computation time when constructing the point cloud since we have the undistorted points readily available for each depth frame.

We temporally and spatially align the latest color camera pose with the depth camera pose for the most accurate depiction of a color point cloud. The device’s long-range depth sensor mode produces a buffer of depth information, which has a maximum frame rate of 5 frames per second (FPS), and for the color camera, we specify a frame rate of 15 FPS to select the nearest red, green, and blue (RGB) image. We create two thread-safe queues to buffer multiple frames of each camera and then, based on the timestamp of each frame and the order in which the frames come in, we grab the pair with the lowest timestamp difference that is currently available. We compress the depth and color images with RVL and JPEG compression and then, together with the head pose and audio of the streamer, send them to the remote server [19].

### 2.2. Server

We implemented a client-server model in Python to transfer data between streamers and receivers. Since emergency situations and training scenarios oftentimes entail sensitive information about patients from a streamer’s point of view, we use the Transmission Control Protocol (TCP) over User Datagram Protocol (UDP) for secure and reliable communication. From the streamer, the server receives a stream of bytes consisting of the undistorted depth and infrared (IR) images, an RGB image of the user’s point of view, and their respective timestamps. We decompress the depth and IR images from the streamer and feed them into the Azure Body Tracking software development kit (SDK) v1.1.0 to segment the color point cloud of the patient out of the environment [20]. In addition to the compressed depth and RGB image pair, the server sends the user’s head pose, audio, and depth mappings to the receiving application. The server also relays the receiver’s hand tracking data, head pose, and audio to show their virtual embodiment to the streamer.

### 2.3. Receiving Application

The primary purpose of the receiving side application allows (1) students to visualize a 3D view of the teacher’s interaction with a patient, or (2) teachers to provide instructions to the student. These scenarios are interchangeable within the same application session, where streamers and receivers can switch roles and stream the alternative 3D perspective. We tested the system on a HoloLens 2 and a PC. However, since it is based on OpenXR, the receiver application should also run on devices such as HoloLens 2, Quest Pro, Windows Mixed Reality, SteamVR, and any other OpenXR-compatible device with only minor modifications. The receiver visualizes streams of data, including head pose, audio, depth camera, and color camera from the remote server, and sends streams of data including head, audio, and hand pose, for immersive telepresence. The receiving application obtains compressed depth and color images that are decompressed and projected as a color point cloud stream of the patient.

For each depth image that arrives, we iterate through the pixels in parallel and validate them through data masking. Each depth image is paired with an RGB image to map color pixel data to depth points. Given Research Mode’s exposed functionality to map image points onto a unit plane one meter from the user, we deproject depth pixels in camera space to 3D points in world space. Then we project these world-mapped depth points into RGB camera space as pixel coordinates. If these pixels fit within the dimensions of the RGB camera, we keep these depth points and assign their color value; otherwise, they are ignored. Once we obtain the array of location and color information, we use Unreal Engine’s Niagara visual effects system for the efficient rendering of a point cloud as a particle system. The receiver sees this virtual representation of the patient in addition to a 3D model of the streamer’s head with respect to the virtual patient.

Together, these components enable the receiver to view a live 3D representation of the streamer interacting in real time with a patient while in separate locations. By seeing the hand and head movements of the teacher next to the virtual patient, the student can follow the teacher’s actions on the real patient and vice versa. Communication between the teacher and the student is done through the AR HMD microphone and speaker. Since the interaction between the teacher and the student is in real time, whenever the student has questions, they can ask the teacher to perform the action again, mimicking in-person instruction.

### 2.4. Experimental Design

To evaluate the system, we conducted a pilot study with five users. A trainer (streamer) was wearing the HoloLens 2 and looking at a person lying on the ground. The trainer performed ten actions on the person on the ground, such as lifting the left arm, poking the nose, simulating CPR or touching an ear. The student (receiver) was viewing the point cloud stream of the trainer’s actions on their own AR device. After each trainer’s action, the student confirmed that they had correctly experienced the action by describing the action in their own words, e.g., “you lifted the left arm” or “you poked the nose”. The trainer counted how many of these actions the student correctly identified. After the experiment, each student was asked 16 questions about the complexity, applicability, realism, and enjoyment of the application.

## 3. Results & Discussion

**Figure 2:**
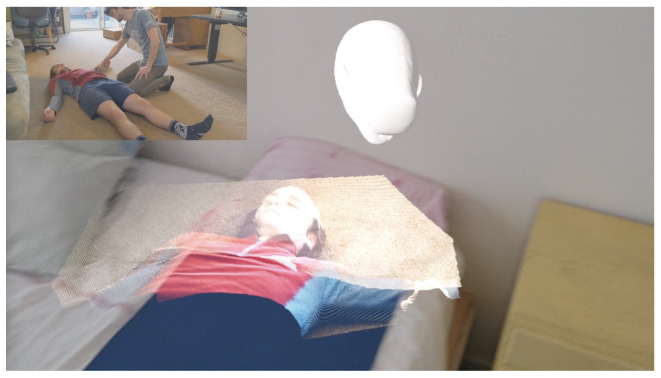
Small image top-left: A trainer (streamer) wearing a HoloLens 2 and looking at a patient. Large image right: A mixed reality capture of the student’s first-person view (receiver) showing the color point cloud of the patient from the perspective of the trainer wearing the HoloLens 2. The white head model represents the head pose of the trainer. A full (video demo, https://youtu.be/oo6A1yKrJFY, accessed on 22 February 2022) also shows the segmentation of the patient.

We successfully developed the system with an overview of the final system in Figure 2. The application can stream the first-person 3D perspective of the streamer in real time to the receiver and allows the receiver to view a 3D point cloud of the streamer’s field of view.

### 3.1. Technical Evaluation

Our high-performance system maintains a frame rate of 60 hertz when accessing all sensor streams on the device. While the user experiences stable holograms, the system efficiently partitions its processing of large amounts of data for synchronization and rendering. Depth sensor frames are streamed at a steady 5 FPS for the long-range mode and are temporally aligned with incoming RGB frames within a 20-millisecond time window. JPEG compression of color images has a 10:1 ratio, and RVL compression of depth and IR images has a 4:1 ratio. The receiver’s hands, rendered as 3D models in the eyes of the streamer, are lightweight enough to sustain a smooth 45 FPS as well. After capturing 1000 depth frames, we measured the improvements in parallelizing the construction of a color point cloud. Our results show that running the algorithm in parallel is 2.4 times faster than an iterative approach, with a time of 17 milliseconds per depth frame.

### 3.2. Pilot Study

**Figure 3:**
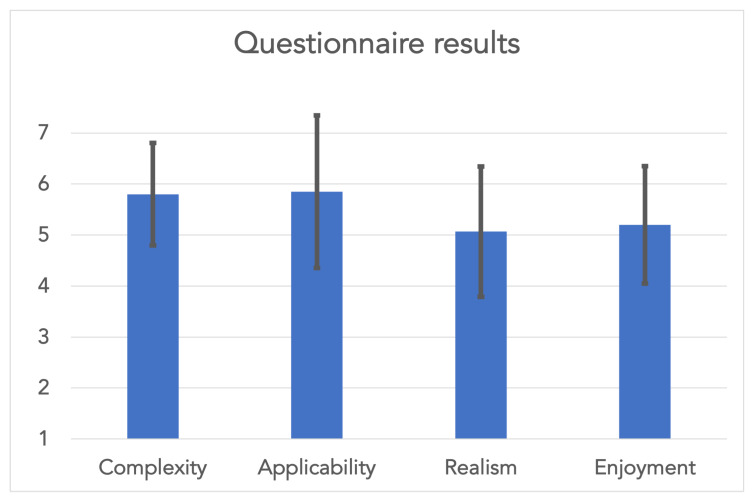
Average ± standard deviation of the questions asked to five users during a preliminary study to rate the complexity (1 difficult, 7 easy), applicability (1 impractical, 7 practical), realism (1 not realistic, 7 very realistic), and enjoyment (1 boring, 7 entertaining) of the application.

Each student was able to correctly identify all actions that the trainer performed on the person lying on the ground. One student had to ask the trainer to repeat two actions because the action was not visible in the field of view of the 3D point cloud. Figure 3 shows the results of the questionnaire. Users rated the complexity of the application with a 5.8 ± 1.0 (1 difficult, 7 easy), the applicability with a 5.9 ± 1.5 (1 impractical, 7 practical), the realism with a 5.1 ± 1.3 (1 not realistic, 7 very realistic), and the enjoyment factor of the application with a 5.2 ± 1.2 (1 boring, 7 entertaining) with all values being the average score ± one standard deviation.

### 3.3. Remote Teaching

Our application enables teachers and students to interact in the same 3D environment while in separate locations. By seeing the 3D perspective and head movements of the teacher, the student can follow the teacher’s actions in the real patient. Alternatively, it is possible to stream the 3D view of the patient from the student to the teacher, allowing the teacher to guide the student. This is a critical use case because healthcare experts are extremely scarce and unevenly distributed around the world, which highly constrains access to them, especially for trainees from low-resource environments. Our application can potentially help increase access to these experts since it does not require students to be collocated with their teachers.

### 3.4. Patching a Remote Specialist

Another potential use case of our application could be the patching of a remote specialist while treating a patient in a low-resource environment similar to what Strak et al. proposed [16]. For example, a paramedic could use our application to quickly and accurately communicate the status of a patient to the physician whose license they are under to receive guidance or permission for modifications to their protocol. Because our work presented would allow the physician to see exactly what the paramedic sees, it could help diagnose the problem, learn how to help, and offer a modified procedure.

### 3.5. Limitations

One limitation of our application is that the receiver cannot decide what they are viewing but only sees what the streamer looks at one RGB-D frame at a time. A potential solution to this could be to have a pointer arrow controlled by the receiver to indicate to the streamer where to look. The accumulation of points by the streamed RGB-D frames could create a more complete virtual representation of the streamer’s environment that the receiver can see even when the streamer looks elsewhere.

Furthermore, the current price of AR devices is another limiting factor. However, as alluded to in our clinical background discussion, we expect this cost to continue to decrease. As AR technology and network coverage continue to improve, we are hopeful that remote AR training and guidance can be leveraged to counter the shortage of medical experts. We believe that our work contributes to this development and will improve the quality and quantity of medical training in low-resource areas in the long term.

## 4. Conclusions

We successfully implemented a complete and cross-platform proof-of-concept AR system that enables remote users to teach and train medical procedures. The highlight of the work presented here lies in the high scalability of our standalone approach, which can be easily set up in remote locations and low-resource settings without expensive medical equipment or external sensors.

## Data Availability

Not applicable.

## Abbreviations

The following abbreviations are used in this manuscript:

AR	Augmented reality
FPS	Frames per second
HMD	Head-mounted display
IR	Infrared
RGB	Red, green, blue
SDK	Software development kit
SLAM	Simultaneous localization and mapping
VR	Virtual reality
WHO	World Health Organization
